# DNA Barcoding and Microsatellites Help Species Delimitation and Hybrid Identification in Endangered Galaxiid Fishes

**DOI:** 10.1371/journal.pone.0032939

**Published:** 2012-03-06

**Authors:** Delphine Vanhaecke, Carlos Garcia de Leaniz, Gonzalo Gajardo, Kyle Young, Jose Sanzana, Gabriel Orellana, Daniel Fowler, Paul Howes, Catalina Monzon-Arguello, Sofia Consuegra

**Affiliations:** 1 Institute of Biological, Environmental and Rural Sciences, Aberystwyth University, Aberystwyth, United Kingdom; 2 Department of BioSciences, College of Science, Swansea University, Swansea, United Kingdom; 3 Laboratorio de Genética, Acuicultura y Biodiversidad, Universidad de Los Lagos, Osorno, Chile; 4 Falkland Islands Fisheries Department, Stanley, Falkland Islands; Biodiversity Insitute of Ontario - University of Guelph, Canada

## Abstract

The conservation of data deficient species is often hampered by inaccurate species delimitation. The galaxiid fishes *Aplochiton zebra* and *Aplochiton taeniatus* are endemic to Patagonia (and for *A. zebra* the Falkland Islands), where they are threatened by invasive salmonids. Conservation of *Aplochiton* is complicated because species identification is hampered by the presence of resident as well as migratory ecotypes that may confound morphological discrimination. We used DNA barcoding (*COI*, cytochrome *b*) and a new developed set of microsatellite markers to investigate the relationships between *A. zebra* and *A. taeniatus* and to assess their distributions and relative abundances in Chilean Patagonia and the Falkland Islands. Results from both DNA markers were 100% congruent and revealed that phenotypic misidentification was widespread, size-dependent, and highly asymmetric. While all the genetically classified *A. zebra* were correctly identified as such, 74% of *A. taeniatus* were incorrectly identified as *A. zebra*, the former species being more widespread than previously thought. Our results reveal, for the first time, the presence in sympatry of both species, not only in Chilean Patagonia, but also in the Falkland Islands, where *A. taeniatus* had not been previously described. We also found evidence of asymmetric hybridisation between female *A. taeniatus* and male *A. zebra* in areas where invasive salmonids have become widespread. Given the potential consequences that species misidentification and hybridisation can have for the conservation of these endangered species, we advocate the use of molecular markers in order to reduce epistemic uncertainty.

## Introduction

Given the current ratio of species discovery to extinction rates, it is likely that many species will go extinct before they are properly described [1]. Accurate classification is thus an essential first step towards effective conservation of local biodiversity [2]–[3]. This is particularly critical in the Southern Hemisphere, where a high proportion of endemic species are poorly known, and are now threatened by non-native introductions [4]. Biological invasions are a leading cause of animal extinctions [5] and one of the main threats to aquatic biodiversity [6], particularly for fishes [7], which tend to display high rates of endemism.

The diversity of fish species in Patagonia is very low, with only five families represented (Galaxiidae, Trichomycteridae, Diplomystidae, Atherinopsidae and Percichthyidae) [8]. The genus *Aplochiton* is one of the three genera representing the family Galaxiidae in South America [9] and has two recognised species *A. zebra* and *A. taeniatus* (Jenyns 1842) restricted to Patagonia [9], and in the case of *A. zebra*, also present in the Falkland Islands [10]. The existence of a third species, *A. marinus*, has been suggested [8]–[9], but its taxonomic status remains unclear [11]. Little is known about the ecology and biogeography of *Aplochiton* [9], [12], which tend to display a patchy, restricted distribution [9], and whose reproductive ecology has only recently been described [13]. *Aplochiton* are believed to have an amphidromous life cycle with a marine larval phase followed by juvenile growth and spawning in freshwater [12], [14], although landlocked populations are also known [12]. Apparent declines in the abundance of *Aplochiton* have been attributed to a number of stressors [15]–[16], most notably predation and/or competition from invasive salmonids, which are widespread and dominate freshwater fish communities throughout much of *Aplochiton*'s range [17]–[20]. *A. zebra* has been classified as ‘in danger of extinction’ in parts of its range, while the conservation status of *A. taeniatus* remains unclear due to data deficiency [17].

Fishes from the family Galaxiidae are morphologically very diverse, particularly in relation to buccal arrangements and shape of caudal fin [21]–[23]. Although both *A. zebra* and *A. taeniatus* have elongate, fusiform bodies with a slender, deeply forked tail [12], they exhibit morphological differences that appear to be related to their different trophic positions. Thus, *A. taeniatus* seems to attain a larger size than *A. zebra* and is considered an specialist that preys mostly on fish, displaying particular adaptations for piscivory such as a large mouth, enlarged teeth and elongated stomach. In contrast, *A. zebra* is considered a generalist that feeds mainly on aquatic invertebrates [12], and resembles more other galaxiids that are also generalized invertebrate predators [17], [24]–[25]. Differences in body size and trophic ecology probably reflect differences in niche breadth, which could ultimately result in different vulnerability to salmonid invasions through predation and resource competition.

Discrimination between *A. zebra* and *A. taeniatus* is currently based on variation in meristic (number of vertebrae, gillrakers and fin rays) and morphological traits (body depth, and relative size of jaw, fins, and head in relation to eye diameter) [11]–[12]. However, phenotypic traits can vary widely among individuals and populations with different life histories [11], particularly in species with diadromous life histories such as *Aplochiton*, making phenotypic based identification unreliable [9]. In addition, marked changes in allometric relationships between juvenile and adult stages of *Aplochiton* may cause taxonomical problems [24]. Thus, in order to establish appropriate conservation measures for these species, clarification of the taxonomic status of *Aplochiton* is urgently needed. To help resolve such conservation challenge, we carried out the molecular analysis of 421 individuals classified as *A. zebra* and 36 individuals classified as *A. taeniatus* based on the morphological characteristics described by previous workers [11]–[12]. We used DNA barcoding, a diagnostic technique based on sequence variation at a small segment of the mitochondrial cytochrome c oxidase I gene (*COI*; [25]), that provides an inexpensive and simple tool for identifying novel species [26], and also for describing cryptic species which are difficult to detect phenotypically [27]–[29]. We sequenced two mitochondrial DNA regions commonly used for fish barcoding (*COI* and *cyt b*; [30]–[31]) to discriminate between *A. zebra* and *A. taeniatus*, and to clarify their distributions in Chilean Patagonia and the Falkland Islands. In addition, we carried for the first time an analysis of genetic diversity and population differentiation of these endangered species, using a set of microsatellite markers that we have recently developed [32].

## Methods

### Study populations

**Figure 1:**
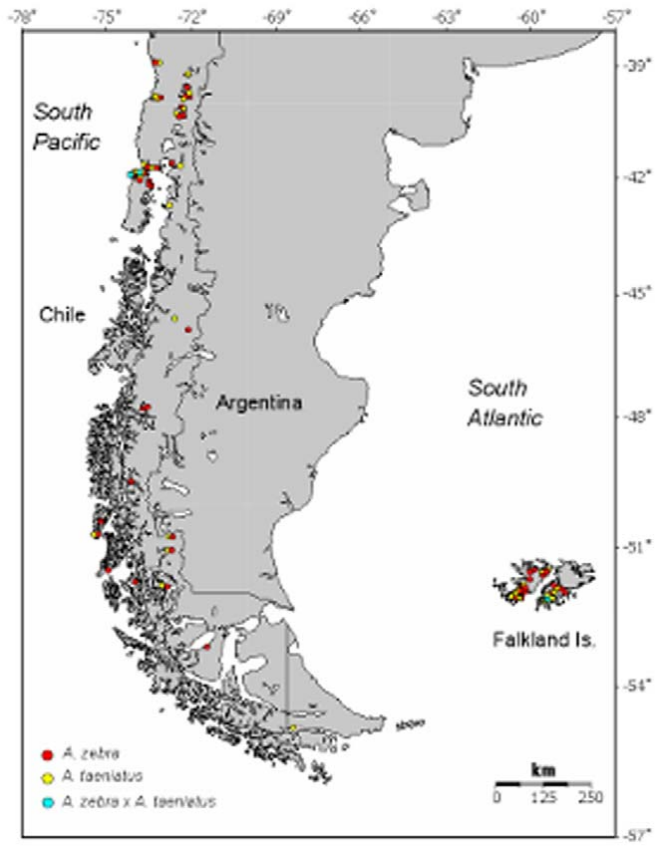
Accepted distribution of the genus *Aplochiton* in Chilean Patagonia and the Falkland Islands, based on data published in [16], [17] (represented by circles) and samples collected during the present study (represented by squares).

*Aplochiton* spp. were collected by electrofishing at 20 different sites in Chilean Patagonia (n = 376) and 15 sites in the Falkland Islands (n = 80; Table 1; Figure 1). Samples from Chile were collected under permit No. 958, 17 April 2008 from the Chilean Subsecretary of Fishing; samples from the Falkland Islands were collected under licence No. R0221, issued by The Falkland Islands Government, Environmental Planning Department. Individuals were identified *in situ* as *A. zebra* or *A. taeniatus* based on body depth, relative size of the head and the caudal peduncle in relation to body length, and body coloration/pigmentation [11]–[12]. Fin clips were preserved in 95% ethanol and stored at 4°C for genetic analysis. We recorded wet weight (*W_t_*, 0.1 g) and either total length (*T_L_*, mm) - measured from the tip of the snout to the tip of the tail, or fork length (*F_L_*, mm) – measure from the tip of the snout to the fork of the tail, depending on country and field crew. To standardise body size measurements obtained by different field crews, we converted total length (*T_L_*) to fork length (*F_L_*) based on the following empirical expression derived from 30 matched samples: *F_L_* = −3.076+0.945 *T_L_* (*R* ^2^ = 0.993, n = 30, *P*<0.001), and used Fulton's condition factor *K* = (*W_T/_F_L_^3^)*×10,000 as a measure of body shape [33]. Size comparisons between locations and species were carried out in SYSTAT v.11.

**Table 1:** Distribution of *Aplochiton zebra*, *A. taeniatus* and hybrids (Hyb), amongst samples collected in Chilean Patagonia and the Falkland Islands.

River	Area	Latitude	Longitude	Date	*A. zebra*	*A. taeniatus*	Hyb	Total
**Chilean Patagonia**								
Blanco-Enco	Mainland	−39.574	−72.149	24-03-09	29	0	0	29
Punahue	Mainland	−39.831	−72.037	24-03-09	21	0	0	21
Quimán	Mainland	−40.113	−72.343	26-03-09	27	0	0	27
Iculpe	Mainland	−40.314	−72.439	31-03-09	30	0	0	30
Pitreño	Mainland	−40.326	−72.319	26-03-09	30	1	0	31
Futangue	Mainland	−40.331	−72.266	30-03-09	30	0	0	30
Lenca	Mainland	−41.605	−72.682	14-04-09	17	0	0	17
U24	Chiloé	−41.811	−74.031	29-11-07	0	1	0	1
U25	Chiloé	−41.814	−73.971	29-11-07	0	1	0	1
Huincha	Chiloé	−41.879	−73.652	18-03-09	1	28	1	30
U26	Chiloé	−41.886	−73.962	30-11-07	0	3	0	3
U27	Chiloé	−41.893	−73.959	30-11-07	0	5	0	5
Punihuil	Chiloé	−41.931	−74.023	01-12-07	0	2	0	2
U28	Chiloé	−41.946	−74.024	02-12-07	22	6	1	29
U29	Chiloé	−41.959	−74.040	03-12-07	21	0	0	21
U30	Chiloé	−41.984	−74.012	04-12-07	0	12	0	12
U34	Chiloé	−42.110	−73.484	08-12-07	16	0	0	16
U17	Chiloé	−42.115	−73.484	04-11-07	20	0	0	20
U33	Chiloé	−42.168	−73.479	07-12-07	25	0	0	25
U20	Chiloé	−42.208	−73.401	09-11-07	26	0	0	26
Total					315	59	2	376
**Falkland Islands**								
North Arm	E. Falkland	NA	NA	2007/08	1	30	0	31
Half-way House	E. Falkland	−51.997	−59.283	2008/09	4	2	0	6
Findlay Creek	E. Falkland	−51.888	−59.025	2008/09	0	2	0	2
N.W. Arm House	E. Falkland	−52.167	−59.487	26-01-09	0	3	0	3
Deep Arroyo	E. Falkland	−51.955	−59.208	28-01-09	0	2	0	2
Bull Pass	E. Falkland	−51.890	−59.007	27-01-09	1	2	0	3
Spots Arroyo	E. Falkland	−52.025	−59.343	27-01-09	4	3	1	8
Fish Creek	W. Falkland	−51.891	−60.368	10-12-08	3	1	0	4
Stewarts Brook	W. Falkland	−52.048	−60.682	01-12-08	0	2	0	2
Gibraltar Stream	W. Falkland	−52.091	−60.331	05-12-08	1	3	0	4
First Arroyo	W. Falkland	−52.083	−60.534	2008/09	7	0	0	7
Outflow L. Sullivan	W. Falkland	−51.792	−60.211	11-12-08	0	2	0	2
Poncho Valley	W. Falkland	−51.973	−60.435	08-12-08	1	1	0	2
Mt Rosalie House	W. Falkland	−51.485	−59.368	21-01-09	0	1	0	1
Red Pond	W. Falkland	−51.557	−59.612	17-12-08	0	3	0	3
Total					22	57	1	80
**Grand total**					**337**	**116**	**3**	**456**

### mtDNA analysis

DNA was extracted using the Wizard® SV 96 DNA Purification Kit following manufacturer's instructions. For mtDNA analysis, regions of the *COI* (cytochrome c oxidase subunit I) and *cyt b* (cytochrome *b*) genes were amplified. A region of 515 bp of the 5′ region of the mitochondrial *COI* gene was amplified for 367 fish using primers FishF1 and FishR1 [30]. In addition, the primers L14724 [34] and H15149 [35] were used to amplify a region of 354 bp of the *cyt b* gene for 105 fish. PCR was conducted using an initial denaturation step at 95°C for 5 minutes, followed by 35 cycles at 94°C for 30 seconds, 55°C for 1 minute, 72°C for 2 minutes and one cycle for a final extension of 10 minutes at 72°C. Double stranded DNA was purified from the PCR using the High-Throughput Wizard® SV 96 PCR Clean-Up System, quantified using the NanoDrop1000 v.3.7 Spectrophotometer (Thermo Fisher Scientific), and both strands were sequenced on an ABI 3100 DNA analyser (Applied Biosystems CA, USA). Sequences were aligned using BioEdit v. 7.0.9 [36] and corrected by eye. *Cyt b* sequences were aligned against the *Aplochiton cyt b* sequence deposited in Genbank [37].

Intraspecific diversity was estimated by the number of haplotypes (H) and nucleotide diversity (π) [38] using DnaSP v.5 [39]. Intra- and interspecific divergence were calculated using the Kimura-2- parameter (K2P) distance [40] in MEGA 4.0 [41].

### Microsatellite analysis

Amplifications were performed for thirteen microsatellite markers (Aze1-Aze13) originally designed for *A. zebra* [32], in three separate multiplex PCR reactions (multiplex1: Aze1, Aze2, Aze3, Aze4, Aze5, Aze6; multiplex 2: Aze8, Aze9 and Aze10; multiplex 3: Aze11, Aze12, Aze13 and Aze14) using the QIAGEN Multiplex PCR kit (QIAGEN, Sussex, UK). Touchdown PCR was performed using an initial denaturing step of 15 min at 95°C followed by 8 cycles of 95°C for 45 s, 64°C −56°C annealing for 90 s and extension at 72°C for 1 min. 25 additional cycles were then performed using an annealing temperature of 56°C and a final extension at 72°C for 10 min. PCR products were resolved on an ABI3130×l sequencer and analyzed using GeneMapper v 4.0 (Applied Biosystems, USA).

Microsatellite loci were examined for evidence of gametic disequilibrium using GENEPOP [42]. FSTAT [43] was used to estimate Hardy Weinberg proportions (HWE), number of alleles (A), observed and expected heterozygosities (Ho and He) and genetic distance among populations (F***_ST_***). Levels of significance were adjusted by sequential Bonferroni correction for multiple tests [44]. Analysis of Molecular Variance (AMOVA) was performed in Arlequin 3.1 [45] in order to estimate the level of genetic variance owed to species differentiation. An UPGMA tree based on Nei's distance [46] was built in TFPGA [47] to provide a graphical representation of the divergence between and within *Aplochiton* species. Statistical confidence on the UPGMA tree nodes was computed by 10,000 bootstrap permutations.

### Hybrid identification

Principal Component Analysis (PCA) based on the multilocus genotypes was carried out in GENETIX [48] in order to separate the species and identify any intermediate genotypes resulting from species admixing [49]. We also used the Bayesian assignment approach implemented in STRUCTURE 2.3.2 [50] assuming K = 2 (burn-in period of 25,000 steps and 100,000 MCMC iterations, 20 runs for each K), applying the admixture model with correlated allele frequencies. The results from the 20 replicates were averaged using the software CLUMPP [51] and the output was represented using DISTRUCT 1.1. Individuals were assigned on the basis of their membership coefficient Q. In order to assess the statistical power of the admixture analysis to detect hybrids, we used HYBRIDLAB [52] to simulate parental and hybrid genotypes. We used 100 *Aplochiton zebra* and 100 *Aplochiton taeniatus* (as classified in STRUCTURE by individual membership values of Q>0.9) to simulate the genotypes of 100 individuals from each of the parental and hybrid classes, repeated 10 times. Given the importance of threshold Q-values for identification of hybrids in STRUCTURE [53], we run the simulated purebred and hybrid individuals in STRUCTURE using an admixture model with no prior information and K = 2 to define the appropriate Q for individual assignment with our set of microsatellites. STRUCTURE was also used to compare the structuring within species. Finally, potential hybrids were checked for the presence of private alleles of *A. zebra* and *A. taeniatus* in their genotypes. Private alleles were defined as occurring only in one species or occurring in both species but with a frequency of less than 1% in one of them to incorporate the possibility of genotype and/or sampling error [54].

## Results

### mtDNA (COI and cyt b) sequence variation

We sequenced a total 367 *Aplochiton* sp., 335 of which had been identified as *A. zebra* and 32 as *A. taeniatus* using phenotypic criteria. All 367 individuals were sequenced for *COI* and 105 of these were also sequenced for *cyt b*.

Based on *COI* sequence variation, the 367 individuals were resolved into two distinct haplogroups consisting of 262 (haplogroup A) and 105 individuals (haplogroup B), respectively (Figure S1). All of the 262 individuals of haplogroup A had been identified as *A. zebra* based on phenotypic criteria, whereas 32 out of the 105 individuals of haplogroup B had been initially identified as *A. taeniatus*. On this basis, we classified fish in haplogroup A as *A. zebra* and fish in haplogroup B as *A. taeniatus*. We detected 6 unique haplotypes defined by 5 mutations amongst *A. zebra* (HA1 n = 237, HA2 n = 5, HA3 n = 6, HA4 n = 9, HA5 n = 4, HA6 n = 1 individual in each case) and 4 haplotypes differing in 3 mutations amongst *A. taeniatus* (HB1 n = 68; HB2 n = 1; HB3 n = 21; HB4 n = 15; GenBank accession numbers HQ540330-HQ540339). Nucleotide diversity was π = 0.00115 ± 0.00011 for *A. taeniatus* and π = 0.00044 ± 0.00008 for *A. zebra*.

For the *cyt b* region, amplification failed in 75 individuals identified as *A. zebra* based on phenotype and *COI* sequence, possibly as a result of mutation in one of the priming sites. Variation in the *cyt b* sequence resolved the remaining 105 individuals into two haplogroups, consisting of 22 and 83 fish respectively. Using the previous *COI* classification, fish in the first group corresponded to *A. zebra* (Haplogroup A), while fish in the second group corresponded to *A. taeniatus* (Haplogroup B). The most common haplotype of *A. taeniatus* showed 100% base agreement with the unique *A. zebra cyt b* haplotype present in GenBank [37], which was derived from a single specimen collected in the Falkland Islands (Waters pers. comm.). This suggests that the sample in Genbank may have been misidentified, given that *A. taeniatus* had not been described in the Falkland Islands previously [10], [17].

We detected 5 *cyt b* unique haplotypes defined by 4 mutations amongst *A. zebra* (HA1 n = 7; HA2 n = 8; HA3 n = 1; HA4 n = 5; HA5 n = 1) and 3 haplotypes differing in 3 mutations amongst *A. taeniatus* (HB1 n = 4; HB2 n = 78; HB3 n = 1; GenBank accession numbers HQ540340-HQ540347). As with *COI*, none of the haplotypes were shared between species. Nucleotide diversity was low for both species: π = 0.00292 ± 0.0004 for *A. zebra* and π = 0.00039 ± 0.00017 for *A. taeniatus*.

### Extent of intraspecific and interspecific divergence

The two species differed in 38 fixed mutations at *COI* and 31 fixed mutations at the 354 bp fragment of *cyt b*, and did not share any haplotypes or mutations in either marker. Intraspecific divergence (K2P distance) at *COI* was 0.0012 ± 0.0002 for *A. zebra* and 0.0015 ± 0.0005 for *A. taeniatus*, while interspecific distance was 70 fold greater (0.0882 ± 0.0142). At *cyt b*, intraspecific K2P divergence was 0.0029±0.0018 for *A. zebra* and 0.0004±0.0003 for *A. taeniatus*. Again, divergence was approximately two orders of magnitude greater between species (0.1005 ± 0.0173) than within species. Furthermore, classification agreement for individuals amplified for both markers was 100%. Thus, both mtDNA markers provided complete and concordant species discrimination.

### Microsatellite analysis

A total of 456 *Aplochiton* samples (367 of which were also sequenced for mtDNA) were genotyped using 13 nuclear microsatellite markers. Results from the admixture analysis conducted in STRUCTURE assuming K = 2 and based on 11 microsatellites (excluding two microsatellites with low amplification success in *A. taeniatus*) separated the samples into three distinct groups: a first group of 338 individuals classified as *A. zebra* by barcoding, a second group of 113 individuals classified as *A. taeniatus* by barcoding, and a third group of five individuals representing potential hybrids (Figure S2).

Eleven of the thirteen microsatellites (85%) originally designed for *A. zebra* reliably amplified in *A. taeniatus* and nine (69%) were polymorphic. Successful cross-amplification was observed for all of the 13 *A. zebra* microsatellites analysed, although two of them (Aze11 and Aze13) amplified only in 33% and 6% of the *A. taeniatus* samples, respectively. Two of the microsatellites, Aze4 (allelic size 99 bp) and Aze14 (allelic size 92 bp) were monomorphic for *A. taeniatus* (Table 2 and Table S1). These alleles were private (Aze14-92) to *A. taeniatus* or present only at a very low frequency in *A. zebra* (1.2%; Aze4-99), and therefore in combination these could be used to discriminate between *A. taeniatus* and *A. zebra*. In total 70% of the alleles were private to one of the two species.

**Table 2:** Sample size (N), allele size ranges, number of alleles (Na), expected heterozygosity (He) and observed heterozygosity (Ho) for the microsatellite markers Aze1-Aze14 for *Aplochiton taeniatus* and *Aplochiton zebra*.

	*A. taeniatus*					*A. zebra*				
Locus	N	Size range	Na	He	Ho	N	Size range	Na	He	Ho
Aze1	99	125–139	5	0.12	0.12	336	121–139	8	0.47	0.44
Aze2	106	127–169	13	0.43	0.26	333	123–169	13	0.76	0.68
Aze3	110	87–89	2	0.15	0.19	337	75–91	5	0.46	0.39
Aze4	108	99	1	0.00	0.00	260	89–115	10	0.54	0.26
Aze5	106	122–217	29	0.91	0.84	333	122–321	88	0.97	0.88
Aze6	109	151–177	10	0.67	0.69	324	157–191	16	0.84	0.70
Aze8	109	201–225	7	0.47	0.29	329	173–307	43	0.94	0.70
Aze9	106	79–175	20	0.90	0.84	324	79–267	45	0.97	0.88
Aze10	110	166–172	4	0.53	0.50	333	154–190	16	0.86	0.78
Aze11	38	124–164	13	0.87	0.49	337	108–172	20	0.69	0.56
Aze12	107	127–253	32	0.80	0.61	323	151–241	40	0.93	0.84
Aze13	7	134–176	6	0.80	0.10	336	124–174	18	0.69	0.63
Aze14	108	92	1	0.00	0.00	337	100–112	7	0.32	0.21

These observed rates of cross-amplification (85%) and polymorphism (69%) fall within the expected range of cross-species microsatellite amplification/polymorphism success, given the evolutionary distance of the two *Aplochiton* species estimated by pairwise *cyt b* genetic distances. Thus, using our estimated rate of *cyt b* divergence between *A. zebra* and *A. taeniatus* (0.1005), the expected rates of amplification and polymorphism would be 84% and 42%, or 56% and 33%, using the relationship found for cetaceans and frogs, respectively [55].

Pairwise population differentiation comparison showed high levels of divergence between both species with F*_ST_* values ranging from 0.24 to 0.32 (*P*<0.001). Analysis of molecular variance (AMOVA) of the two species of *Aplochiton* (excluding hybrids) showed that 25.2% of genetic variance was distributed between species (F*_ST_* = 0.300; *P*<0.001), while 4.8% of the variance was due to differences between populations within species (F*_SC_* = 0.065; *P*<0.001), and 70% of the variance was due to variation among individuals within populations (F*_CT_* = 0.252; *P*<0.001). Average observed heterozygosity (excluding the Az13 locus) and allelic richness were lower for *A. taeniatus* (*H*_o_ = 0.37; *Ar* = 3.5) than for *A. zebra* (*H*_o_ = 0.61; *Ar* = 2.5).

**Figure 2:**
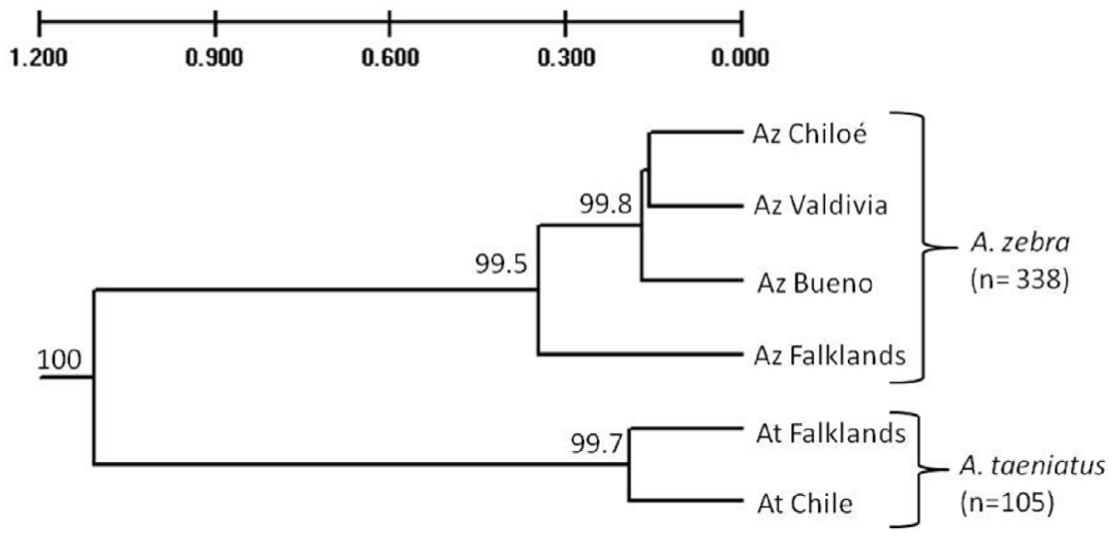
UPGMA clustering of *A. zebra* and *A. taeniatus* based on microsatellite markers using Nei's original distance [**46**]. Numbers at each branch node represent % bootstrap support derived from 1,000 replicates.

**Figure 3:**
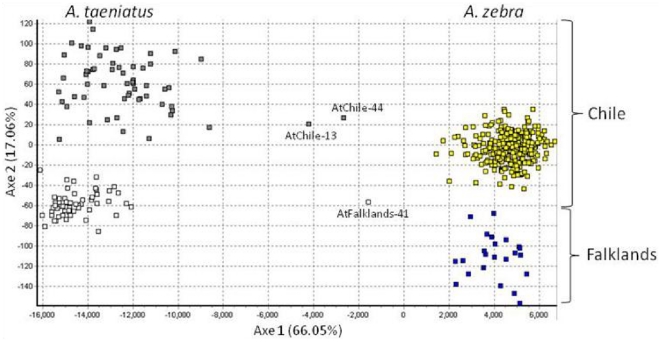
Species discrimination and identification of hybrids based on Principal Component Analysis of *Aplochiton taeniatus* and *Aplochiton zebra* microsatellite genotypes. PC1 and PC2 represent the first two factorial components. Three putative F_1_ hybrids are indicated by their ID sample codes.

The UPGMA tree clustered the individuals in two main groups supported by high bootstrap values (higher than 99%). This is in agreement with results from mtDNA analyses and revealed some further regional structuring (Figure 2). In order to compare the relative structuring of both species, F*_ST_* analyses of genetic distance were carried out among populations within species when the sample size allowed it. Among populations of *A. zebra* in Chile, the estimated genetic distance was F*_ST_* = 0.045 but a similar analysis could not be carried out in the Falklands due to the limited number of individuals per population (Table 1). Genetic distance for *A. taeniatus* in the Falklands was estimated by grouping the individuals regionally in East and West Falklands (F*_ST_* = 0.039 *P*<0.001), while for Chile only the two populations with enough *A. taeniatus* (R. Huincha and U30) were compared (F*_ST_* = 0.010 *P* = 0.025). Genetic differentiation between Chilean Patagonia and the Falkland Islands was highly significant for both species (Figures 2, 3), being greater for *A. taeniatus* (F*_ST_* = 0.135, *P*<0.0010) than for *A. zebra* (F*_ST_* = 0.084, *P*<0.001).

### Identification of hybrids

Principal Component Analysis (PCA) based on microsatellite genotypes of individual *Aplochiton* revealed a clear segregation between *Aplochiton* species and between geographical regions within species (Figure 3). Three individuals were identified as hybrids, and of them had mtDNA haplotypes typical of *A. taeniatus* (both for *cyt b* and *COI*), but microsatellite alleles unique to *A. zebra*. Results from admixture analysis using STRUCTURE and assuming K = 2 assigned 451 individuals as *A. taeniatus* or *A. zebra* while 5 individuals showed genotypes admixed between both clusters (Figure S2). *A. zebra* membership coefficients (Q1) ranged between 0.84 and 0.99 (mean: 0.99±0.02) and *A. taeniatus* membership coefficients (Q2) ranged from 0.89 to 0.99 (mean: 0.99±0.01). Pure individuals from each species with Q>0.9 were used to simulate parental and hybrid classes in HYBRIDLAB. Using STRUCTURE, the simulated parental classes (*A. zebra* and *A. taeniatus*) were correctly assigned to their species (Figure S2) with a minimum Q threshold of 0.88 (average Q = 0.96). F_1_ hybrids were also correctly identified with Qmax = 0.67 and so were the F_2_ hybrids and backcrosses showing a Qmax = 0.79.

None of the hybrids showed a multilocus genotype which could be classified as parental. However, we did not observe strong differences in Q values for the different hybrid classes (averages Qmin/max = 0.46–0.54). On this basis, we identified three potential F_1_ hybrids from the admixture analysis that corresponded to those individuals previously classified as hybrids by PCA analysis, with the following relative admixture values: Q1/Q2 = 0.37/0.63; Q1/Q2 = 0.60/0.40; and Q1/Q2 = 0.67/0.33 (Figure S2). Two further individuals were identified by STRUCTURE (but not by PCA) as potential hybrids, one with *A. taeniatus* mtDNA (AtChile-36) with admixture value Q1/Q2 = 0.26/0.74 and a second with *A. zebra* mtDNA (AzU29-36) and admixture coefficients Q1/Q2 = 0.84/0.16 (Figure S2). Based on their Q values, these fish could represent F_2_ or backcrosses, but results from HYBRIDLAB suggest that our combination of microsatellites did not allow for accurate discrimination.

The three F_1_ hybrids all possessed *A. taeniatus* mtDNA and showed between four and six clearly introgressed microsatellite alleles (Table 3). Two of the three hybrids had been phenotypically identified as *A. zebra*, and one as *A. taeniatus*. Thirty per cent of the alleles were shared between species. The three identified hybrids possessed two *A. taeniatus* alleles fixed for Aze 4 (99) and Aze 14 (92), and were heterozygous for Aze 14 (92) and one of the *A. zebra* private alleles (108). Two of the hybrids (ATChile-44 and ATFalklands-41) were homozygous for the *A. taeniatus* characteristic 99 allele (Aze 14) that only appears at low frequency in *A. zebra* (1.34%), and were homozygous for alleles private to *A. zebra* for one microsatellite locus (Aze 10; ATFalklands-41) and two microsatellite loci (Aze 2 and Aze 10; ATChile-44), respectively. The remaining hybrid was heterozygous for private alleles of each species in five of the eleven markers, and had shared alleles in the remaining six markers.

**Table 3:** Population frequencies of *A. zebra* alleles present in the three hybrids with *A. taeniatus* mitochondrial DNA haplotype.

Locus	Allele	*A. taeniatus* (%)	*A. zebra* (%)
Aze2	127	0.47	40.57
	129	0	14.67
Aze4	103	0	56.51
Aze6	173	0	11.84
Aze10	174	0	5.53
	178	0	15.86
	180	0	18.26
Aze12	183	0.91	11.88
Aze14	108	0	78.4

### Phenotypic misidentification

The incidence of phenotypic misidentification was relatively high (85/454 = 19%) and, perhaps more importantly, highly asymmetric (Table 4; McNemar symmetry test χ^2^ = 85, df = 1, *P*<0.001). Thus, whereas all the 339 fish genetically classified as *A. zebra* were correctly identified as such based on their phenotype, most (85/115 or 74%) of the *A. taeniatus* were wrongly identified as *A. zebra*. Misidentification was particularly evident in the case of the Falkland Islands, where only one species (*Aplochiton zebra*) was thought to exist.

**Table 4:** Classification of matched samples of *Aplochiton zebra* and *Aplochiton taeniatus* based on morphometric traits (phenotype) and molecular markers (*COI*, cytochrome *b* and microsatellites).

Classification by molecular markers	Identification by phenotypic criteria			
	*A. zebra*	*A. taeniatus*	Total	% agreement
*A. zebra*	338	0	338	100
*A. taeniatus*	85	30	115	26.1
Hybrids	2	1	3	
Total	426	31	456	
% agreement	79.6	96.8		

### Distributional range and abundance of *A. zebra* and *A. taeniatus*

Our results indicate that the two *Aplochiton* species occur sympatrically in Chilean Patagonia and also in the Falkland Islands, although their relative abundances differed significantly across sample sites (Table 1; G-test = 183.59, df = 3, *P*<0.001). In Chile, *A. taeniatus* appears to be more abundant on the Island of Chiloé (98% of individuals), whereas *A. zebra* was more abundant in mainland samples (58% of individuals; Fisher exact test, *P*<0.001). Likewise, in the Falkland Islands, the relative abundance of the two species appears to differ significantly between the two islands (Fisher exact test, *P* = 0.014): *A. taeniatus* appears to be the dominant species in East Falkland (81%), whereas the two species appear to be equally common in West Falkland (52% vs. 48%).

**Figure 4:**
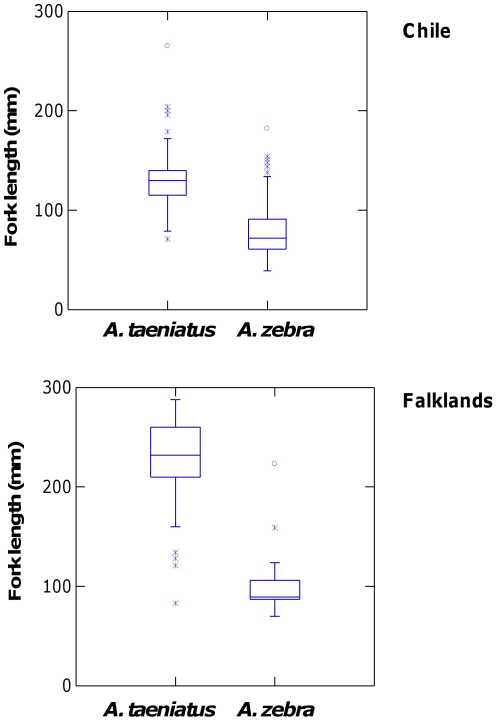
Size variation (fork length, mm) of *A. taeniatus* and *A. zebra* in Chilean Patagonia and the Falkland Islands, as inferred from molecular identification.

*A. taeniatus* was significantly larger than *A. zebra* in both Chile and the Falklands (Figure 4; Species effect *F*_1,441_ = 233.4, *P*<0.001), though the size difference was more pronounced in the Falkland Islands than in Chile (Species x Location interaction *F*_1,441_ = 38.39, *P*<0.001). *Aplochiton* in the Falklands were significantly larger than in Chile, regardless of species identity (Location effect *F*_1,441_ = 4.62, *P* = 0.032). Analysis of condition factor indicates that *A. taeniatus* has a thinner, more streamlined body than *A. zebra* (*F*_1,435_ = 23.5, *P*<0.001), regardless of location (*F*_1,435_ = 0.93, *P* = 0.334).

## Discussion

By using two different mtDNA markers commonly employed for species barcoding, our phylogenetic reconstruction of *Aplochiton* reveals two distinct, non-overlapping haplogroups corresponding to *A. zebra* and *A. taeniatus*. The average intraspecific distance among individuals was 0.12% for *A. zebra* and 0.15% for *A. taeniatus*, compared to 8.8% between the two *Aplochiton* species. The observed divergence is thus 60–100 times higher between groups than among individuals within groups, supporting the contention that *A. zebra* and *A. taeniatus* are indeed two different species, and that DNA barcoding correctly identified them as such [30].

Furthermore, results using 11 microsatellite markers were fully consistent with the groups previously identified by DNA barcoding.

The observed ratio of intra to interspecific divergence in *Aplochiton* is very similar to that reported for many other fish species across several families [30], [56], where the average intraspecific distance was 0.39% and the average interspecific distance was 8.11–9.93%. On the other hand, we found no evidence to support the existence of a third species (*A. marinus*), whose presence in Chilean Patagonia has been suggested by some workers [8]–[9], [16], [57]. Although our Chilean samples were collected from 20 different locations, the sites were largely concentrated in the northern part of the species' range. With this caveat in mind, we suggest that molecular evidence is needed to clarify the taxonomic status of *A. marinus*. As indicated by McDowall [17], [24]–[25], it is possible that *A. marinus* is simply the migratory form of *A. taeniatus*.

Our data suggest that misidentification of *Aplochiton* could be common. Indeed, 19% of *A. taeniatus* in our study were misidentified as *A. zebra* based on phenotypic traits, suggesting that *A. taeniatus* could occur as two ecotypes, one which is readily identified by workers in the field, and another, more cryptic form, which is often mistaken for *A. zebra*. In contrast, all individuals classified as *A. zebra* using genetic data were correctly identified as such using phenotypic traits. Despite the threat that invasive salmonids pose to the conservation of *Aplochiton* [17]–[19], it is not clear to what extent the two species are equally vulnerable to salmonid invasions, or whether one species has been more impacted than the other. Both species appear to occupy similar fast-flowing habitats [16]–[17], but may have different diets, as *A. taeniatus* is thought to have a specialised piscivorous diet, while *A. zebra* appears to feed mostly on invertebrates. Our results indicate that *A. taeniatus* tends to have a more streamlined body and attain a larger size than *A. zebra*, which is consistent with previous findings [17], [24] and a greater dependence on piscivory, as reported initially by McDowall & Nakaya [12]. As invasion success in fish often depends on invader body size [58], a larger body size may make *A. taeniatus* more resilient than *A. zebra* to salmonid invasions, but its more specialised diet may also make it a more vulnerable to competition from ecologically similar salmonids.

Whatever the precise nature of salmonid impacts, misidentification could have important consequences for the conservation of endangered *Aplochiton*, if it leads to inappropriate protection measures or fails to recognise the species' distinct needs [59]. Misidentification can also have important implications for *ex-situ* conservation [60], as captive breeding could inadvertently produce hybrids and impact on the very same species targeted for conservation.

DNA barcoding in our study provides the first evidence of *A. taeniatus* in the Falkland Islands, and shows that the species is more widely distributed than previously thought, being present in both East and West Falkland. Molecular data also suggest that *A. zebra* might be less common - and its distribution more restricted - than reported in recent studies [16]. The two *Aplochiton* species appear to occur sympatrically across their entire range (Figure 1) and our study provides clear evidence, for the first time, that they also hybridise in the wild. Three hybrids were detected by both PCA and admixture analyses, and based on their membership coefficients (Q), these are most likely F_1_ hybrids. Two further hybrids were also identified by admixture analyses, but simulations carried out with HYBRIDLAB could not unambiguously resolve their origin as either F_2_ or backcrosses. If we consider only the unambiguous F_1_ hybrids identified by both PCA and STRUCTURE, the presence of *A. taeniatus* mtDNA indicates that the direction of hybridisation was in all cases via female *A. taeniatus* and male *A. zebra*. Such asymmetric hybridisation could have resulted from prezygotic barriers [61]–[62], postzygotic effects [63]–[64] or a combination of both [65]. Given that *A. zebra* will normally be smaller than *A. taeniatus*, this may have facilitated sneaking behaviour by male *A. zebra* during reproduction, leading to asymmetrical hybridisation, as observed in other fish species ([66]). Asymmetrical hybridisation can also arise from differences in the relative abundance of parental species, with the less common species typically becoming the female parent [67], and from Dobzhansky-Muller incompatibilities, whereby reciprocal interspecific crosses produce different rates of fertilization and/or sterility [68].

Although the low number of hybrids in our study precludes further testing of potential causes of *Aplochiton* hybridisation, the hypothesis that the less abundant species provides the female parent does not appear to be consistent with the data. Thus, *A. zebra* was dominant in one river, *A. taeniatus* in another river, and the two species were found in roughly the same proportions in the third river where hybrids were detected. When gene flow varies amongst hybridising species, interspecific introgression is more likely to occur in the more fragmented species [69]. In that sense, the degree of differentiation between Chilean and Falklands populations was greater for *A. taeniatus* than for *A. zebra*, but the structuring of Chilean *A. zebra* was similar in magnitude to the structuring of *A. taeniatus* within the Falklands, and hybrids occurred in both regions. Further studies, particularly in the Falkland Islands, are needed in order to clarify the roles of population fragmentation and mating behaviour on asymmetrical hybridisation in *Aplochiton*. Sexing would also reveal whether the two *Aplochiton* species conform to Haldane's rule, whereby the heterogametic sex is usually absent, rare, or sterile amongst F_1_ hybrids [70], and whether populations exhibit fluctuating sex ratios, as found in other Salmoniformes [71].

Recent work suggests that hybridisation is relatively frequent in animals, even if it tends to occur at low rates [72] and is typically less widespread than among plants. Hybridisation can play an important role in species' evolution [73], either enhancing or reducing the adaptive persistence of hybridising species. Thus, interspecific gene introgression could increase the genetic diversity and evolutionary potential of hybrids, while outbreeding depression could render them unviable or infertile [74]. Anthropogenic stressors, such as environmental degradation or introduction of exotic species, have been found to increase hybridisation rates, though the mechanisms can be subtle and not readily apparent. For example, increased water turbidity in Lake Victoria seems to have disrupted visually-mediated mate choice and reproductive isolation among cichlids [75], while stocking with hatchery-reared fish may have facilitated salmonid hybridisation in the Iberian peninsula [76]. Hybridisation can also occur when one species expands into the other species' range and there are no reproductive barriers [49]. In this sense, it is unclear what role, if any, exotic salmonids may have played in *Aplochiton* hybridisation. Invasive salmonids are widespread in Chilean Patagonia [77] and the Falkland Islands [10], where they displace and outcompete native galaxiids [17]–[20], but whether they may have facilitated *Aplochiton* hybridisation by increasing secondary contact [78] is not clear.

Like most galaxiids, the biology and conservation needs of *Aplochiton* are poorly known [17], and this probably constitutes one of the biggest obstacles to their conservation [18]. Almost 20% of galaxiids have only been identified over the last 25 years, and in most cases their conservation status has either not been evaluated (NE −55%) or suffers from data deficiency (DD −14%). Given that most of the remaining galaxiids are listed by the IUCN Red List as being critically endangered (CR, 8%), or vulnerable (VU, 18%), the implications of taxonomic misclassification could be serious because under such data deficient scenarios management may be acting upon the wrong species. Such uncertainty, termed epistemic uncertainty [79], results from lack of knowledge and represents a property of the observer, and therefore extrinsic to the scientific problem being addressed. Our study illustrates how molecular markers can help to decrease epistemic uncertainty in the identification of *Aplochiton*, paving the way for more efficient conservation programmes.

In summary, we show for the first time that the two *Aplochiton* species occur in sympatry and hybridise in Chilean Patagonia and also in the Falkland Islands, where only *A. zebra* was thought to be present and where our study indicates that *A. taeniatus* might in fact be the most common species. We also show that some microsatellite markers are diagnostic for *Aplochiton*, and provide a first estimate of genetic diversity and regional differentiation for these species. Finally, we reveal through DNA barcoding that phenotypic misidentification is common and caution against sole reliance on morphological traits for species delimitation of *Aplochiton* and other poorly known galaxiid fishes.

## Supporting Information

Figure S1 **Nucleotide sequence alignment of mitochondrial DNA of** ***Aplochiton zebra*** **(A) and** ***Aplochiton taeniatus*** **(B) according to (a)** ***COI*** **haplogroups and (b)** ***Cyt b*** **haplogroups.**
(DOC)

Figure S2 **Hybrid assignments based on (a) simulated membership proportions of 100 multilocus genotypes per class using HYBRIDLAB and (b) results of admixture analysis using STRUCTURE:** ***A. zebra*** **parentals,** ***A. taeniatus*** **parentals, F_1_ hybrids, F_2_ hybrids and backcrosses.** Results from the admixture analysis in STRUCTURE are for K = 2, averaged from 20 runs. Each bar constitutes an individual genotype. Y-axis represents the proportion of each individual attributable to each cluster, which can be deduced from the colour of the bars. The horizontal line represents the upper limit for pure bred individuals estimated with HYBRYDLAB. Pure *A. zebra* genotypes are represented in pale grey; pure *A. taeniatus* genotypes are represented in dark grey. Potential hybrids are identified by an asterisk (*).
(DOC)

Table S1 **Microsatellite allele frequencies per locus per species (Aze1-Aze4).**
(DOC)
