# Supplementary material for: DNA Barcoding and Microsatellites Help Species Delimitation and Hybrid Identification in Endangered Galaxiid Fishes
Source: PLoS One. 2012 Mar 6;7(3):e32939. doi: 10.1371/journal.pone.0032939 (PMC3295793; doi:10.1371/journal.pone.0032939)
Supplement: Figure S1 — Nucleotide sequence alignment of mitochondrial DNA of Aplochiton zebra (A) and Aplochiton taeniatus (B) according to (a) COI haplogroups and (b) Cyt b haplogroups. (DOC) [file pone.0032939.s001.doc]

**Figure S1.** Nucleotide alignment of mitochondrial DNA sequences of the two Aplochiton species. (a) COI haplogroups: A (*A. zebra*) and B (*A. taeniatus*) and (b) Cyt b haplogroups: A (*A. zebra*) and B (*A. taeniatus*).

**(a)**

10 20 30 40 50 60 70 80 90

....|....|....|....|....|....|....|....|....|....|....|....|....|....|....|....|....|....|

**Az_HA1** **CAGGGTCAAAGAAGGTTGTATTCAGGTTTCGGTCCGTGAGTAGTATGGTGATCCCGGCAGCAAGGACAGGGAGAGATAAGAGGAGGAGCA**

**Az_HA2** **..........................................................................................**

**Az_HA3** **....................................................T.....................................**

**Az_HA4** **..........................................................................................**

**Az_HA5** **..........................................................................................**

**Az_HA6** **..........................................................................................**

**At_HB1** **.............A..C.....T..............A.....C..A.....T...........A..T.....G..C.....C.......**

**At_HB2** **.............A..C.....T..............A.....C..A.....T...........A..T.....G..C.....C.......**

**At_HB3** **.............A..C.....T..............A.....C..A.....T...........A..T.....G..C.....C.......**

**At_HB4** **.............A..C.....T...........T..A.....C..A.....T...........A..T.....G..C.....C.......**

100 110 120 130 140 150 160 170 180

....|....|....|....|....|....|....|....|....|....|....|....|....|....|....|....|....|....|

**Az_HA1** **CGGCCGTAATTAGCACGGCCCATACAAATAGCGGCGTTTGGTATTGGGAAATTGCGGGGGGTTTCATGTTAATAATGGTCGTGATAAAGT**

**Az_HA2** **..........................................................................................**

**Az_HA3** **..........................................................................................**

**Az_HA4** **..........................................................................................**

**Az_HA5** **..........................................................................................**

**Az_HA6** **..........................................................................................**

**At_HB1** **....T.................C........G..T..C.....C..............................................**

**At_HB2** **....T.................C........G..T..C.....C..............................................**

**At_HB3** **....T.................C........G..T..C.....C..............................................**

**At_HB4** **....T.................C........G..T..C.....C..............................................**

190 200 210 220 230 240 250 260 270

....|....|....|....|....|....|....|....|....|....|....|....|....|....|....|....|....|....|

**Az_HA1** **TAATGGCCCCTAAGATGGAAGAAATGCCGGCCAGGTGAAGAGAAAAAATAGTGAGGTCCACAGAGGCACCAGCGTGCGCTAGATTGCCTG**

**Az_HA2** **........................................................................................C.**

**Az_HA3** **..........................................................................................**

**Az_HA4** **..................................A.......................................................**

**Az_HA5** **..........................................................................................**

**Az_HA6** **..........................................................................................**

**At_HB1** **............GA..A..............T.A.....................A...........G..............G.....C.**

**At_HB2** **............GA..A..............T.A.....................A...........G..............G.....C.**

**At_HB3** **............GA..A..............T.A.....................A...........G..............G.....C.**

**At_HB4** **............GA..A..............T.A.....................A...........G..............G.....C.**

280 290 300 310 320 330 340 350 360

....|....|....|....|....|....|....|....|....|....|....|....|....|....|....|....|....|....|

**Az_HA1** **CCAATGGGGGGTACACCGTTCATCCTGTGCCAGCCCCTGCTTCAACCCCAGAAGAGGCAAGCAAGAGCAGGAAAGAGGGTGGGAGAAGTC**

**Az_HA2** **..........................................................................................**

**Az_HA3** **..........................................................................................**

**Az_HA4** **..........................................................................................**

**Az_HA5** **.T........................................................................................**

**Az_HA6** **..............................................T...........................................**

**At_HB1** **...G.........T........C.....T......................................G...........G..A.....C.**

**At_HB2** **...G.........T........C.....T........C.............................G...........G..A.....C.**

**At_HB3** **...G.........T........C.....T......................................G...........G..A.....C.**

**At_HB4** **...G.........T........C.....T......................................G...........G..A.....C.**

370 380 390 400 410 420 430 440 450

....|....|....|....|....|....|....|....|....|....|....|....|....|....|....|....|....|....|

**Az_HA1** **AGAAGCTCATATTGTTCATCCGAGGGAATGCCATATCCGGGGCCCCGATCATAAGTGGAATTAATCAGTTCCCAAAGCCCCCAATCATAA**

**Az_HA2** **..........................................................................................**

**Az_HA3** **..........................................................................................**

**Az_HA4** **..........................................................................................**

**Az_HA5** **..........................................................................................**

**Az_HA6** **..........................................................................................**

**At_HB1** **................T....................G....................................................**

**At_HB2** **................T....................G....................................................**

**At_HB3** **................T....................G.....T..............................................**

**At_HB4** **................T....................G....................................................**

460 470 480 490 500 510

....|....|....|....|....|....|....|....|....|....|....|....|....|

**Az_HA1** **TTGGCATGACTATAAAAAAGATTATCACGAAAGCGTGTGCCGTGACGATAACATTATAAATCTGG**

**Az_HA2** **.................................................................**

**Az_HA3** **.................................................................**

**Az_HA4** **.................................................................**

**Az_HA5** **.................................................................**

**Az_HA6** **.................................................................**

**At_HB1** **.......A.........................................................**

**At_HB2** **.......A.........................................................**

**At_HB3** **.......A.........................................................**

**At_HB4** **.......A.........................................................**

**(b)**

10 20 30 40 50 60 70 80 90

....|....|....|....|....|....|....|....|....|....|....|....|....|....|....|....|....|....|

**Az_HA1** **TCCAACATTTCTGTATGATGAAATTTTGGCTCTCTCCTAGGCCTATGCTTGGCAAGCCAGATTCTTACGGGACTCTTTCTTGCTATGCAC**

**Az_HA2** **..........................................................................................**

**Az_HA3** **.............................T............................................................**

**Az_HA4** **..........................................................................................**

**Az_HA5** **A.........................................................................................**

**At_HB1** **.....T...................................G...........G......G.............A.....C..C......**

**At_HB2** **.....T...................................G...........G......G.............A.....C..C......**

**At_HB3** **.....T...................................G...........G......G.............A.....C..C......**

100 110 120 130 140 150 160 170 180

....|....|....|....|....|....|....|....|....|....|....|....|....|....|....|....|....|....|

**Az_HA1** **TACACCTCCGATATTTCCACCGCCTTTTCCTCCGTCACCCATATCTGCCGAGATGTCAGCTACGGCTGGCTTATCCGAAGCATGCATGCC**

**Az_HA2** **..........................................................................................**

**Az_HA3** **..........................................................................................**

**Az_HA4** **..........................................................................................**

**Az_HA5** **..........................................................................................**

**At_HB1** **.................T...........T..............T...........T..............A.....G..T.........**

**At_HB2** **.................T...........T..............T...........T..............A.....G..T.........**

**At_HB3** **.................T...........T..............T...........T..............A.....G..T.........**

190 200 210 220 230 240 250 260 270

....|....|....|....|....|....|....|....|....|....|....|....|....|....|....|....|....|....|

**Az_HA1** **AACGGCGCATCCTTTTTCTTCATCTGTATTTACATGCATATCGGCCGAGGCCTTTACTATGGCTCGTATCTTTATAAAGAGACCTGAAGC**

**Az_HA2** **................................................................................A.........**

**Az_HA3** **..........................................................................................**

**Az_HA4** **...............................................G................................A.........**

**Az_HA5** **................................................................................A.........**

**At_HB1** **..T.............................T.....C...........G.....T.................C.....A.......A.**

**At_HB2** **..T.............................T.....C...........G.....T........A........C.....A.......A.**

**At_HB3** **..T.............................T..A..C...........G.....T........A........C.....A.......A.**

280 290 300 310 320 330 340 350

....|....|....|....|....|....|....|....|....|....|....|....|....|....|....|....|....

**Az_HA1** **ATCGGCGTAGTTCTACTCCTCCTCGTTATGATGACTGCCTTTGTTGGCTATGTCCTCCCCTGAGGACAAATATCATTCTGAGGG**

**Az_HA2** **....................................................................................**

**Az_HA3** **....................................................................................**

**Az_HA4** **....................................................................................**

**Az_HA5** **....................................................................................**

**At_HB1** **.....G..G.....G..T.....T.....A..A...........C........T..T...........................**

**At_HB2** **.....G..G.....G..T.....T.....A..A...........C........T..T...........................**

**At_HB3** **.....T..G.....G..T.....T.....A..A...........C........T..T...........................**
