# Supplementary material for: DNA Barcoding and Microsatellites Help Species Delimitation and Hybrid Identification in Endangered Galaxiid Fishes
Source: PLoS One. 2012 Mar 6;7(3):e32939. doi: 10.1371/journal.pone.0032939 (PMC3295793; doi:10.1371/journal.pone.0032939)

**Figure S2.**

1. Membership proportions of 100 multilocus genotypes per class simulated using HYBRIDLAB and results of the admixture analyses using STRUCTURE: *A. zebra* parentals, *A. taeniatus* parentals, F1 hybrids, F2 hybrids and backcrosses.

1. Identification of hybrids based on admixture analysis. Results from the admixture analysis in STRUCTURE for K = 2, averaged from 20 runs. Each bar constitutes an individual genotype. Y-axis represents the proportion of each individual attributable to each cluster, which can be deduced from the colour of the bars. The horizontal line represents the upper limit for pure bred individuals estimated with HYBRYDLAB. Pure A. zebra genotypes are represented in pale grey; pure A. taeniatus genotypes are represented in dark grey. Potential hybrids are identified with (*)


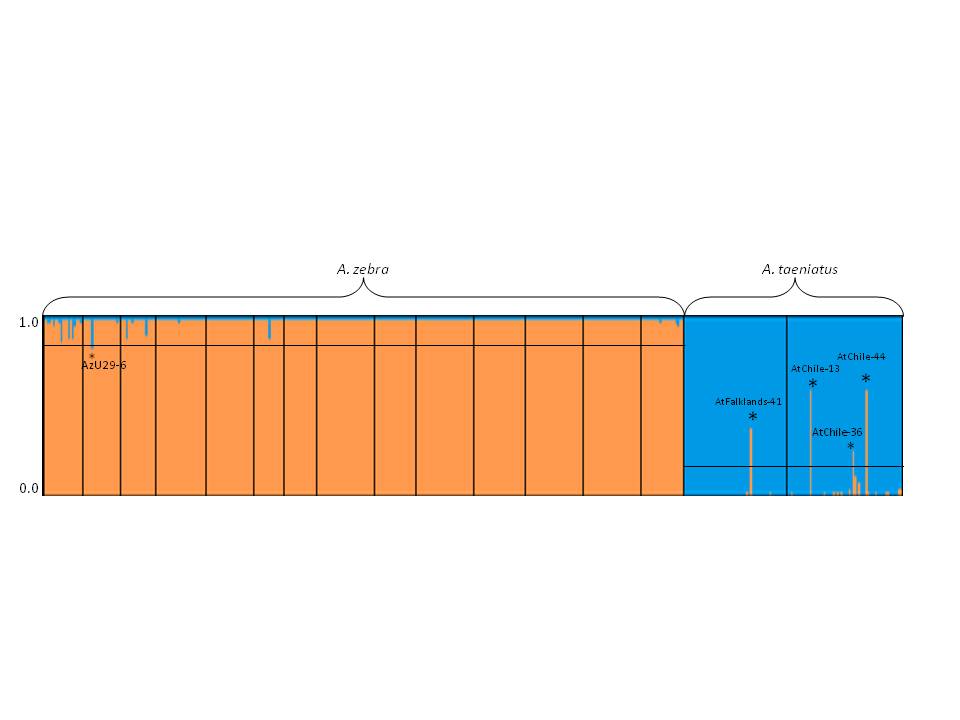

Supplement: Figure S2 — Hybrid assignments based on (a) simulated membership proportions of 100 multilocus genotypes per class using HYBRIDLAB and (b) results of admixture analysis using STRUCTURE: A. zebra parentals, A. taeniatus parentals, F1 hybrids, F2 hybrids and backcrosses. Results from the admixture analysis in STRUCTURE are for K = 2, averaged from 20 runs. Each bar constitutes an individual genotype. Y-axis represents the proportion of each individual attributable to each cluster, which can be deduced from the colour of the bars. The horizontal line represents the upper limit for pure bred individuals estimated with HYBRYDLAB. Pure A. zebra genotypes are represented in pale grey; pure A. taeniatus genotypes are represented in dark grey. Potential hybrids are identified by an asterisk (*). (DOC) [file pone.0032939.s002.doc]
