# Supplementary material for: DNA Barcoding and Microsatellites Help Species Delimitation and Hybrid Identification in Endangered Galaxiid Fishes
Source: PLoS One. 2012 Mar 6;7(3):e32939. doi: 10.1371/journal.pone.0032939 (PMC3295793; doi:10.1371/journal.pone.0032939)
Supplement: Table S1 — Microsatellite allele frequencies per locus per species (Aze1-Aze4). (DOC) [file pone.0032939.s003.doc]

**Table S1.** Microsatellite allele frequencies per locus per species (Aze1-Aze4)

| **Aze1** | AT | AZ | **Aze2** | AT | AZ | **Aze3** | AT | AZ | **Aze4** | AT | AZ |
| --- | --- | --- | --- | --- | --- | --- | --- | --- | --- | --- | --- |
| **121** | 0.00 | 0.30 | **123** | 0.00 | 1.80 | **75** | 0.00 | 0.59 | **89** | 0.00 | 0.19 |
| **123** | 0.00 | 1.04 | **125** | 0.00 | 0.75 | **83** | 0.00 | 0.45 | **99** | 100.00 | 1.34 |
| **125** | 94.00 | 66.62 | **127** | 0.47 | 40.57 | **87** | 12.95 | 70.33 | **101** | 0.00 | 2.11 |
| **127** | 2.50 | 25.52 | **129** | 0.00 | 14.67 | **89** | 87.05 | 26.71 | **103** | 0.00 | 56.51 |
| **129** | 1.00 | 2.23 | **131** | 0.47 | 20.96 | **91** | 0.00 | 1.93 | **105** | 0.00 | 25.09 |
| **133** | 2.00 | 2.97 | **133** | 0.47 | 0.60 |  |  |  | **107** | 0.00 | 4.02 |
| **137** | 0.00 | 0.15 | **135** | 2.80 | 4.34 |  |  |  | **109** | 0.00 | 0.96 |
| **139** | 0.50 | 1.19 | **137** | 0.47 | 0.45 |  |  |  | **111** | 0.00 | 2.68 |
|  |  |  | **139** | 0.47 | 0.00 |  |  |  | **113** | 0.00 | 5.74 |
|  |  |  | **141** | 1.40 | 0.15 |  |  |  | **115** | 0.00 | 1.34 |
|  |  |  | **143** | 0.47 | 6.14 |  |  |  |  |  |  |
|  |  |  | **145** | 8.88 | 8.98 |  |  |  |  |  |  |
|  |  |  | **147** | 77.57 | 0.45 |  |  |  |  |  |  |
|  |  |  | **149** | 0.47 | 0.00 |  |  |  |  |  |  |
|  |  |  | **151** | 5.60 | 0.00 |  |  |  |  |  |  |
|  |  |  | **169** | 0.47 | 0.15 |  |  |  |  |  |  |

**Table S1**. continued (Aze5)

| **Aze5** | AT | AZ |  | AT | AZ |  | AT | AZ |  | AT | AZ |  | AT | AZ |
| --- | --- | --- | --- | --- | --- | --- | --- | --- | --- | --- | --- | --- | --- | --- |
| **122** | 3.74 | 0.15 | **159** | 4.67 | 1.20 | **204** | 0.00 | 0.90 | **236** | 0.00 | 0.30 | **269** | 0.00 | 1.95 |
| **127** | 1.40 | 0.00 | **161** | 0.00 | 1.05 | **207** | 0.00 | 0.45 | **238** | 0.00 | 0.75 | **270** | 0.00 | 0.15 |
| **129** | 0.93 | 0.60 | **163** | 5.61 | 1.50 | **209** | 0.00 | 2.25 | **240** | 0.00 | 1.20 | **272** | 0.00 | 0.90 |
| **131** | 0.47 | 0.00 | **166** | 0.00 | 0.15 | **211** | 0.00 | 0.45 | **242** | 0.00 | 1.05 | **274** | 0.00 | 0.90 |
| **133** | 7.47 | 0.00 | **168** | 12.62 | 4.34 | **212** | 0.47 | 0.75 | **245** | 0.00 | 0.75 | **275** | 0.00 | 0.15 |
| **135** | 0.00 | 1.95 | **170** | 0.47 | 0.15 | **214** | 0.00 | 0.90 | **246** | 0.00 | 0.30 | **277** | 0.00 | 0.15 |
| **136** | 0.00 | 4.34 | **172** | 1.87 | 1.35 | **217** | 0.47 | 0.75 | **247** | 0.00 | 1.35 | **278** | 0.00 | 0.15 |
| **137** | 4.21 | 0.00 | **173** | 7.94 | 2.25 | **218** | 0.00 | 0.15 | **248** | 0.00 | 0.75 | **279** | 0.00 | 0.60 |
| **138** | 1.87 | 0.15 | **175** | 0.00 | 0.75 | **219** | 0.00 | 0.75 | **249** | 0.00 | 0.75 | **283** | 0.00 | 1.50 |
| **140** | 0.00 | 0.15 | **177** | 3.27 | 0.30 | **221** | 0.00 | 2.10 | **250** | 0.00 | 0.30 | **284** | 0.00 | 0.15 |
| **142** | 14.95 | 1.50 | **178** | 0.93 | 0.15 | **223** | 0.00 | 1.05 | **252** | 0.00 | 1.35 | **287** | 0.00 | 0.75 |
| **143** | 2.34 | 1.20 | **180** | 0.00 | 1.35 | **224** | 0.00 | 0.15 | **254** | 0.00 | 0.60 | **288** | 0.00 | 0.90 |
| **145** | 0.00 | 0.15 | **183** | 0.93 | 0.00 | **226** | 0.00 | 1.20 | **255** | 0.00 | 2.10 | **289** | 0.00 | 0.15 |
| **146** | 0.00 | 2.25 | **185** | 0.00 | 0.75 | **227** | 0.00 | 0.30 | **257** | 0.00 | 0.75 | **293** | 0.00 | 0.15 |
| **147** | 0.93 | 7.49 | **190** | 0.00 | 0.90 | **228** | 0.00 | 0.30 | **258** | 0.00 | 3.59 | **296** | 0.00 | 0.15 |
| **149** | 4.67 | 0.60 | **192** | 0.93 | 0.00 | **230** | 0.00 | 0.45 | **260** | 0.00 | 0.90 | **303** | 0.00 | 0.60 |
| **152** | 6.54 | 6.74 | **193** | 1.87 | 0.00 | **231** | 0.00 | 0.60 | **262** | 0.00 | 2.84 | **316** | 0.00 | 0.15 |
| **153** | 1.40 | 0.00 | **195** | 0.00 | 2.99 | **233** | 0.00 | 1.20 | **264** | 0.00 | 0.60 | **321** | 0.00 | 0.15 |
| **154** | 0.93 | 0.00 | **198** | 0.47 | 0.00 | **234** | 0.00 | 0.15 | **265** | 0.00 | 0.15 |  |  |  |
| **157** | 5.61 | 3.59 | **199** | 0.00 | 1.95 | **235** | 0.00 | 0.15 | **267** | 0.00 | 3.14 |  |  |  |

**Table S1.** continued (Aze6-Aze8)

| **Aze6** | AT | AZ | **Aze8** | AT | AZ |  | AT | AZ |  | AT | AZ |
| --- | --- | --- | --- | --- | --- | --- | --- | --- | --- | --- | --- |
| **151** | 0.45 | 0.00 | **173** | 0.00 | 0.15 | **223** | 0.00 | 3.79 | **261** | 0.00 | 0.15 |
| **157** | 0.00 | 0.31 | **175** | 0.00 | 0.15 | **225** | 0.45 | 0.45 | **265** | 0.00 | 0.15 |
| **159** | 46.36 | 4.77 | **181** | 0.00 | 0.15 | **227** | 0.00 | 3.48 | **267** | 0.00 | 2.27 |
| **161** | 1.36 | 1.69 | **189** | 0.00 | 0.15 | **229** | 0.00 | 11.81 | **271** | 0.00 | 0.15 |
| **163** | 33.18 | 2.46 | **193** | 0.00 | 3.94 | **231** | 0.00 | 3.18 | **287** | 0.00 | 0.15 |
| **165** | 5.00 | 0.92 | **195** | 0.00 | 3.79 | **233** | 0.00 | 5.45 | **289** | 0.00 | 6.06 |
| **167** | 1.82 | 22.00 | **197** | 0.00 | 2.58 | **235** | 0.00 | 5.30 | **291** | 0.00 | 0.61 |
| **169** | 8.18 | 2.46 | **199** | 0.00 | 8.18 | **237** | 0.00 | 3.33 | **293** | 0.00 | 0.30 |
| **171** | 1.82 | 18.92 | **201** | 4.09 | 1.52 | **239** | 0.00 | 6.81 | **295** | 0.00 | 1.36 |
| **173** | 0.00 | 11.84 | **203** | 20.45 | 0.45 | **241** | 0.00 | 1.21 | **305** | 0.00 | 0.45 |
| **175** | 0.45 | 4.77 | **205** | 66.36 | 0.91 | **243** | 0.00 | 4.55 | **307** | 0.00 | 0.76 |
| **177** | 1.36 | 16.62 | **207** | 6.36 | 0.00 | **245** | 0.00 | 7.27 |  |  |  |
| **179** | 0.00 | 3.23 | **209** | 1.82 | 0.30 | **247** | 0.00 | 2.73 |  |  |  |
| **181** | 0.00 | 2.46 | **211** | 0.45 | 0.00 | **249** | 0.00 | 0.61 |  |  |  |
| **183** | 0.00 | 6.92 | **217** | 0.00 | 0.91 | **251** | 0.00 | 1.67 |  |  |  |
| **185** | 0.00 | 0.46 | **219** | 0.00 | 0.91 | **255** | 0.00 | 0.30 |  |  |  |
| **191** | 0.00 | 0.15 | **221** | 0.00 | 1.36 | **259** | 0.00 | 0.15 |  |  |  |

**Table S1.** continued (Aze9)

| **Aze9** | AT | AZ |  | AT | AZ |  | AT | AZ |
| --- | --- | --- | --- | --- | --- | --- | --- | --- |
| **79** | 0.46 | 2.31 | **143** | 0.00 | 4.32 | **211** | 0.00 | 0.77 |
| **83** | 0.93 | 0.00 | **147** | 0.93 | 2.16 | **219** | 0.00 | 1.54 |
| **87** | 11.11 | 0.46 | **151** | 0.46 | 5.71 | **223** | 0.00 | 1.70 |
| **91** | 3.24 | 5.40 | **155** | 0.00 | 6.63 | **227** | 0.00 | 1.23 |
| **95** | 15.74 | 7.87 | **159** | 0.00 | 3.86 | **231** | 0.00 | 1.23 |
| **99** | 6.02 | 0.46 | **163** | 1.39 | 3.24 | **235** | 0.00 | 1.85 |
| **103** | 3.24 | 3.09 | **167** | 0.00 | 2.47 | **239** | 0.00 | 2.01 |
| **107** | 15.28 | 3.86 | **171** | 0.00 | 2.78 | **243** | 0.00 | 2.31 |
| **111** | 14.81 | 3.09 | **175** | 0.46 | 1.54 | **247** | 0.00 | 3.40 |
| **115** | 5.56 | 3.86 | **179** | 0.00 | 2.16 | **251** | 0.00 | 2.31 |
| **119** | 8.80 | 3.09 | **183** | 0.00 | 0.31 | **255** | 0.00 | 1.23 |
| **123** | 1.85 | 0.93 | **187** | 0.00 | 0.77 | **259** | 0.00 | 0.31 |
| **127** | 4.63 | 1.70 | **195** | 0.00 | 0.31 | **263** | 0.00 | 0.31 |
| **131** | 1.39 | 1.39 | **199** | 0.00 | 0.93 | **267** | 0.00 | 0.15 |
| **135** | 0.93 | 2.62 | **203** | 0.00 | 0.15 |  |  |  |
| **139** | 2.78 | 1.39 | **207** | 0.00 | 0.77 |  |  |  |

**Table S1.** continued (Aze10-Aze11)

| **Aze10** | AT | AZ | **Aze11** | AT | AZ |  | AT | AZ |
| --- | --- | --- | --- | --- | --- | --- | --- | --- |
| **154** | 0.00 | 0.75 | **108** | 0.00 | 0.44 | **148** | 5.26 | 0.00 |
| **162** | 0.00 | 4.04 | **110** | 0.00 | 0.15 | **150** | 1.32 | 0.00 |
| **164** | 0.00 | 1.05 | **112** | 0.00 | 0.30 | **152** | 1.32 | 0.44 |
| **166** | 52.70 | 0.30 | **118** | 0.00 | 0.44 | **154** | 10.53 | 0.44 |
| **168** | 44.14 | 1.50 | **122** | 0.00 | 0.89 | **156** | 6.58 | 1.33 |
| **170** | 2.25 | 3.74 | **124** | 5.26 | 54.73 | **158** | 3.95 | 0.00 |
| **172** | 0.90 | 9.58 | **126** | 0.00 | 11.53 | **160** | 2.63 | 0.30 |
| **174** | 0.00 | 5.53 | **128** | 0.00 | 7.84 | **164** | 2.63 | 0.00 |
| **176** | 0.00 | 16.76 | **130** | 0.00 | 9.02 | **172** | 0.00 | 0.30 |
| **178** | 0.00 | 15.86 | **132** | 1.32 | 5.33 |  |  |  |
| **180** | 0.00 | 18.26 | **134** | 48.68 | 0.74 |  |  |  |
| **182** | 0.00 | 8.68 | **136** | 0.00 | 1.33 |  |  |  |
| **184** | 0.00 | 6.43 | **138** | 5.26 | 0.00 |  |  |  |
| **186** | 0.00 | 3.89 | **140** | 0.00 | 0.15 |  |  |  |
| **188** | 0.00 | 1.65 | **142** | 5.26 | 3.11 |  |  |  |
| **190** | 0.00 | 1.95 | **144** | 0.00 | 1.18 |  |  |  |

**Table S1.** continued (Aze12-Aze14)

| **Aze12** | AT | AZ |  | AT | AZ |  | AT | AZ | **Aze13** | AT | AZ | **Aze14** | AT | AZ |
| --- | --- | --- | --- | --- | --- | --- | --- | --- | --- | --- | --- | --- | --- | --- |
| **127** | 0.45 | 0.00 | **173** | 1.36 | 7.41 | **211** | 0.00 | 1.39 | **124** | 0.00 | 1.04 | **92** | 100.00 | 0.00 |
| **129** | 2.73 | 0.00 | **175** | 0.00 | 6.17 | **213** | 0.91 | 0.93 | **126** | 0.00 | 0.30 | **100** | 0.00 | 0.15 |
| **133** | 0.91 | 0.00 | **177** | 2.27 | 2.93 | **215** | 0.45 | 0.46 | **128** | 0.00 | 1.19 | **102** | 0.00 | 0.15 |
| **135** | 8.18 | 0.00 | **179** | 1.36 | 5.09 | **219** | 0.45 | 0.77 | **132** | 0.00 | 1.48 | **104** | 0.00 | 0.74 |
| **137** | 19.54 | 0.00 | **181** | 0.00 | 11.72 | **223** | 0.00 | 0.15 | **134** | 42.86 | 52.67 | **106** | 0.00 | 0.30 |
| **139** | 34.09 | 0.00 | **183** | 0.91 | 11.88 | **225** | 0.00 | 0.15 | **136** | 14.29 | 9.20 | **108** | 0.00 | 78.40 |
| **141** | 1.36 | 0.00 | **185** | 0.91 | 2.93 | **229** | 0.00 | 0.15 | **138** | 14.29 | 3.41 | **110** | 0.00 | 19.52 |
| **145** | 3.18 | 0.00 | **187** | 0.45 | 6.02 | **231** | 0.00 | 0.31 | **152** | 0.00 | 0.15 | **112** | 0.00 | 0.74 |
| **147** | 0.45 | 0.00 | **189** | 0.91 | 4.32 | **233** | 0.00 | 0.15 | **156** | 0.00 | 1.48 |  |  |  |
| **151** | 1.36 | 0.62 | **191** | 0.00 | 2.31 | **235** | 0.00 | 0.93 | **158** | 0.00 | 1.48 |  |  |  |
| **155** | 2.27 | 0.15 | **193** | 0.00 | 1.08 | **241** | 0.00 | 0.31 | **160** | 7.14 | 2.23 |  |  |  |
| **157** | 0.00 | 2.78 | **195** | 0.45 | 1.23 | **253** | 0.45 | 0.00 | **162** | 14.29 | 4.30 |  |  |  |
| **159** | 0.91 | 0.15 | **197** | 0.45 | 3.40 |  |  |  | **164** | 0.00 | 5.19 |  |  |  |
| **161** | 1.36 | 2.47 | **199** | 0.91 | 1.85 |  |  |  | **166** | 0.00 | 11.86 |  |  |  |
| **163** | 0.91 | 2.01 | **201** | 0.00 | 0.46 |  |  |  | **168** | 0.00 | 2.23 |  |  |  |
| **165** | 2.27 | 1.39 | **203** | 0.00 | 2.93 |  |  |  | **170** | 0.00 | 0.30 |  |  |  |
| **167** | 1.82 | 2.47 | **205** | 0.00 | 0.31 |  |  |  | **172** | 0.00 | 1.04 |  |  |  |
| **169** | 3.64 | 2.01 | **207** | 0.00 | 0.77 |  |  |  | **174** | 0.00 | 0.45 |  |  |  |
| **171** | 2.27 | 7.25 | **209** | 0.00 | 0.15 |  |  |  | **176** | 7.14 | 0.00 |  |  |  |
